# Bootstrapping Security Policies for Wearable Apps Using Attributed Structural Graphs

**DOI:** 10.3390/s16050674

**Published:** 2016-05-11

**Authors:** Ana I. González-Tablas, Juan E. Tapiador

**Affiliations:** Department of Computer Science, Universidad Carlos III de Madrid, 28911 Leganés, Madrid, Spain; jestevez@inf.uc3m.es

**Keywords:** smartphone security and privacy, security policy, wireless body area networks, policy bootstrapping

## Abstract

We address the problem of bootstrapping security and privacy policies for newly-deployed apps in wireless body area networks (WBAN) composed of smartphones, sensors and other wearable devices. We introduce a framework to model such a WBAN as an undirected graph whose vertices correspond to devices, apps and app resources, while edges model structural relationships among them. This graph is then augmented with attributes capturing the features of each entity together with user-defined tags. We then adapt available graph-based similarity metrics to find the closest app to a new one to be deployed, with the aim of reusing, and possibly adapting, its security policy. We illustrate our approach through a detailed smartphone ecosystem case study. Our results suggest that the scheme can provide users with a reasonably good policy that is consistent with the user’s security preferences implicitly captured by policies already in place.

## 1. Introduction

Security and privacy challenges in wireless body area networks (WBAN), particularly in those equipped with smart devices that can run third-party apps, are greater than in traditional computing and networking scenarios. Many of such devices incorporate numerous sensors that could leak highly sensitive information [1], including the user’s location or sounds and video from his or her surroundings. Most of these aspects have been neglected in the current generation of smart devices, which has caused an alarming escalation in the number and sophistication of security incidents targeting these platforms. For example, recent surveys show that the majority of health and fitness apps for smartphones present significant privacy risk levels, including nonexistent privacy policies [2,3].

According to a 2014 report by Yahoo! [4], users have an average of 95 apps installed on their phones, 35 of which are used on a daily basis; each month, a user accesses around 30 apps; and each app requires four permissions on average. An additional complication comes from the fact that although users declare to be worried about privacy issues, they usually behave inconsistently regarding their privacy concerns [5] or simply do not understand or have the ability to configure privacy policies appropriately [6,7]. Related to this, Bettini and Riboni have recently identified two main challenges related to the user experience when protecting privacy in pervasive systems [8]: providing flexible and adaptable protection mechanisms and capturing user’s preferences without forcing him or her to go through complex settings configurations. Furthermore, the automatic (or, at least, guided) identification of privacy preferences is suggested as a much needed approach to address these challenges.

The problem of helping users to define security and privacy policies for their apps is more acute in the case of wearable technologies. Information sharing among different devices and apps is a central feature of WBANs, hence the criticality of having consistent policies deployed in different apps. Consider, for example, a user whose privacy preferences include providing access to cardiac information to medical apps only. Since different devices may have the ability to provide cardiac data, it must be guaranteed that only medical apps get access to such data in those devices and that they do not share it with non-medical apps.

### 1.1. Overview of Our Contribution

In this work, we address the problem of bootstrapping security and privacy policies for newly-deployed apps in a WBAN composed of smart devices, such as smartphones, smart bracelets and wristwatches, smart glasses and other wearable and implantable medical devices. The overall goal is to provide users with a “reasonably good” policy obtained through a sound procedure that guarantees high consistency with the user’s security preferences. Such a policy could be used as-is, though ideally, it should be provided to the user for additional refinement. Our approach assumes that the user already has a number of properly-configured apps deployed in the WBAN.

As a first contribution, we introduce a framework to model a WBAN as an undirected graph $G_{S}$ whose vertices correspond to devices, apps and app resources, and edges model structural relationships among them. This graph is then augmented with attributes capturing each element’s main features and user-defined tags, resulting in an attributed graph *G*. The framework presented in this paper allows one to model WBANs in terms of attributed structural graphs, which facilitates reusing standard similarity metrics defined for them.

As a second contribution, given a new app *a* to be deployed in the WBAN, we discuss various procedures for finding the app $a^{*}$ closest to *a* in the graph *G*. Specifically, we propose two app similarity metrics that take a random walk approach to compute the similarity between vertices of the graph. Each metric is based on a different construction of the augmented structural graph. The first metric, denoted *ALG-2*, is based on a custom modification to the WBAN setting of the technique proposed in [9]. The second metric, denoted *ALG-3*, further extends this approach by enriching the augmented structural graph in a novel way. Because of the way that *G* is built, similarity is computed considering not only the structural closeness, but also a number of desirable features, including the similarity of the devices in which both apps are deployed, some of the app’s intrinsic features (e.g., its category and requested permissions) and the possible connections through tags and other attributes.

Our final goal is suggesting that the WBAN owner use the security policy of the most similar app as the candidate policy for the newly-deployed app. This seems reasonable insofar as the similarity measure among apps will do its job properly, since a user would likely specify similar privacy preferences for similar apps. More sophisticated schemes based on the same idea are possible, for instance, by deriving a combined security policy from the *k* most similar apps. This is straightforward when using the security policy model introduced later in Section 2.3.

As a third contribution, we demonstrate the similarity metrics *ALG-2* and *ALG-3* on a detailed case study. To do this, we first describe the case study data and specify the WBAN scenario. We then construct the associated structural graph and identify relevant attributes to produce the augmented graph. The doubly-augmented graph is also built by selecting appropriate similarity metrics for each attribute type and tag, adding edges accordingly. Finally, both similarity metrics *ALG-2* and *ALG-3* are computed for each pair of vertices of the augmented and doubly-augmented graph, respectively, and the results are analyzed.

### 1.2. Organization

The rest of this paper is organized as follows. Section 2 introduces our system model for WBANs and illustrates the concept of “security policy” with two examples based, respectively, on ciphertext policy attribute-based encryption (CP-ABE) and attribute-based Android permissions constraint specification. In Section 3, we introduce two similarity metrics for apps based on different constructions of the augmented WBAN structural graph. Section 4 provides the results of applying the two similarity metrics on a detailed use case based on a typical WBAN. In Section 5, we discuss related works in the areas of graph-based similarities and privacy preference learning for smartphone apps. Finally, Section 6 concludes the paper by summarizing our main contributions and discussing its limitations and open problems for future research.

## 2. System Model

We next describe our graph-based model for WBANs composed of interconnected devices, apps and resources supporting user-defined tags and attributes. As a motivating example for our automatic policy-deriving framework, we also introduce a data-sharing scheme for WBAN apps mediated by a classical attribute-based policy.

### 2.1. WBAN Elements

We consider a scenario where a single user *u* owns a set of mobile (and possibly wearable) devices $D=D\left(u\right)=\left\{d_{1},\ldots ,d_{\left|D\right|}\right\}$. Devices can communicate with each other via some wireless communication technology, such as Bluetooth or Wi-Fi, forming a WBAN around user *u*. We do not assume that all devices are permanently connected to the WBAN. While some of them may be actually implanted in the user’s body (e.g., a sensor under the skin or a pacemaker), others will be just worn occasionally (e.g., a sport wristband, a smart watch or a pair of smart glasses). We assume that joining and leaving the WBAN is controlled by the underlying networking technology, for instance via Bluetooth pairing.

Devices may vary from very simple and lightweight instruments, such as a pedometer, to powerful devices, like a smartphone or a tablet. Nonetheless, we require that at least one app can be deployed on each device, which in turn should provide at least one communication interface with other devices (not only with the user, if this is the case). Devices that do not satisfy this requirement will not be considered devices, but sensors. Sensors will be usually paired to a more powerful device in the WBAN and managed through a specific app deployed in such a device.

We assume that each device $d_{i}\in D$ hosts a set of apps $A\left(d_{i}\right)=\left\{a_{i,1},\ldots ,a_{i,|A(d_{i})|}\right\}$. Apps may have been provided by default with the device or else installed by the user. The set A(u)=A of apps that a user has in the WBAN is $A={⋃}_{i=1}^{\left|D\right|}{⋃}_{j=1}^{\left|A(d_{i})\right|}a_{i,j}$. We further assume that each app $a_{i,j}\in A\left(d_{i}\right)$ uses a set $R(a_{i,j})$ of resources that we mainly identify with data storage, where $R\left(a_{i,j}\right)=\left\{r_{i,j,1},\ldots ,r_{i,j,\left|R(a_{i,j})\right|}\right\}$. Data storage may be of several types, such as formatted files/databases or just raw (unstructured) data. The set R(u)=R of resources present in the WBAN is $R={⋃}_{i=1}^{\left|D\right|}{⋃}_{j=1}^{\left|A(d_{i})\right|}{⋃}_{k=1}^{\left|R(a_{i,j})\right|}r_{i,j,k}$.

Apps make use of a platform-provided API to access the capabilities offered by the device. For example, in a regular smartphone, apps can request access to the camera and microphone embedded in the device, get the current location or access the user’s list of contacts. Additionally, although apps are usually executed isolated from other apps running on the same device, it is common that they exploit inter-app communication features provided by the platform to access data and services offered by other apps. For the sake of simplicity, in this paper, we will not consider app interactions that are decided at run time, such as Android intents. However, because of their relevance in current wearable technologies, we will explicitly model apps that are deployed on different devices, but that are strongly paired, in the sense that they have their resources synchronized (e.g., the app deployed in a smartwatch and the associated one running in a smartphone).

**Figure 1:**
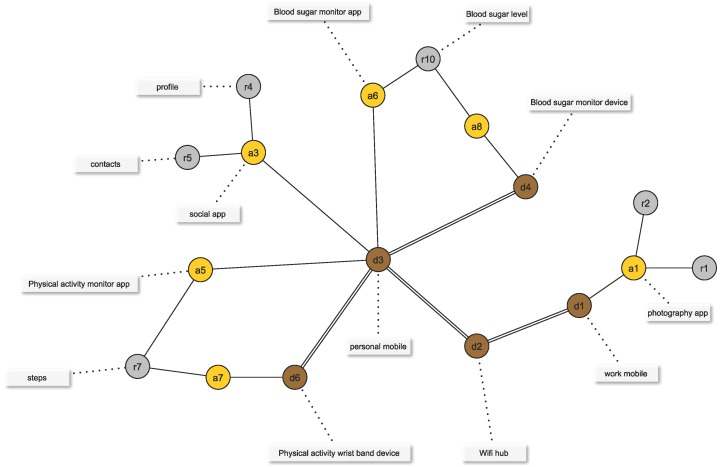
Example of a structural graph of a WBAN containing devices, apps, resources and their relations. Attributes of each element are also shown, but are not part of the structural graph.

All of the elements of our WBAN model may be represented by a structural graph $G_{S}=\left(V_{S},E_{S}\right)$, where $V_{S}=D\cup A\cup R$ is the set of vertices and $E_{S}\subseteq \left(D\times D\right)\cup \left(D\times A\right)\cup \left(A\times R\right)$ is the set of edges that model structural relations between the WBAN elements described above (see Figure 1). The set $E_{S}$ is composed of three different types of relations:

- If there exists a network connection between devices $d_{i}$ and $d_{j}$, then $\left(d_{i},d_{j}\right)\in E_{S}$.
- If app $a_{j}$ is deployed on device $d_{i}$, then $\left(d_{i},a_{j}\right)\in E_{S}$.
- If the app $a_{i}$ makes use of resource $r_{j}$, either because it is owned by $a_{i}$ or because it is a data storage that is shared (synchronized) between $a_{i}$ and another paired app (see, for example, resource $r_{7}$ in Figure 1), then $\left(a_{i},r_{j}\right)\in E_{S}$.

### 2.2. Attributes

In our WBAN model, each device, app or resource is characterized by a set of attributes. We distinguish two types of attributes:

- *Features*, which model a factual characteristic of an element.
- *Tags*, which are used to describe personal (*i.e.*, user-defined) characteristics.

For example, resources corresponding to data storage may be labeled with objective attributes (features) that reflect the type of data stored, such as health related (e.g., blood oxygen level, sweat quantity and content), activity related (e.g., number of steps, heart rate, covered distance), content related (searched terms, visited web sites), banking/financial information, social/business/professional attributes, and so on. App features may include the category it belongs to, the system resources it accesses (for instance, in Android devices, this can be easily captured by the set of permissions the app requests in its manifest) and its content classification, among others. Similarly, a device *d* can be characterized by a set of objective attributes, such as its operating system, its storage and processing capabilities or its amount of battery.

The set of features associated with a device will be denoted as $\hat{D}=\left\{\delta_{1},\ldots ,\delta_{|\hat{D}|}\right\}$. Each attribute $\delta_{j}$ takes values from a domain $Dom\left(\delta_{j}\right)=\left\{\delta_{j,1},\ldots ,\delta_{j,|Dom(\delta_{i})|}\right\}$. The set of features associated with an app and a resource are defined similarly: $\hat{A}=\left\{\alpha_{1},\ldots ,\alpha_{|\hat{A}|}\right\}$, with $Dom\left(\alpha_{j}\right)=\left\{\alpha_{j,1},\ldots ,\alpha_{j,|Dom(\alpha_{j})|}\right\}$; and $\hat{R}=\left\{\rho_{1},\ldots ,\rho_{|\hat{R}|}\right\}$, with $Dom\left(\rho_{j}\right)=\left\{\rho_{j,1},\ldots ,\rho_{j,|Dom(\rho_{j})|}\right\}$. By grouping all features together in a set $F=\hat{D}\cup \hat{A}\cup \hat{R}$, each element $v_{i}\in V_{S}$ can be provided with a feature vector $\left[f_{1}\left(v_{i}\right),\ldots ,f_{N}\left(v_{i}\right)\right]$, where $f_{j}\left(v_{i}\right)\in Dom\left(f_{j}\right)=\left\{f_{j,1},\ldots ,f_{j,|Dom(f_{j})|}\right\}$ is the value taken by the *j*-th attribute. The total number of features is $N=|\hat{D}|+|\hat{A}|+|\hat{R}|$.

The set of user-defined tags will be denoted $T=\left\{t_{1},\ldots ,t_{\left|T\right|}\right\}$. A user can associate an arbitrary subset of tags T(v)⊆T to a node $v\in V_{S}$. For implementation purposes, we will label each element of the WBAN with a tag vector $\left[t_{1}\left(v_{i}\right),\ldots ,t_{\left|T\right|}\left(v_{i}\right)\right]$, this being a binary vector where $t_{j}\left(v_{i}\right)=1$ if tag *j* has been associated with element $v_{i}$ and $t_{j}\left(v_{i}\right)=0$ otherwise.

### 2.3. Security Policy Model

**Figure 2:**
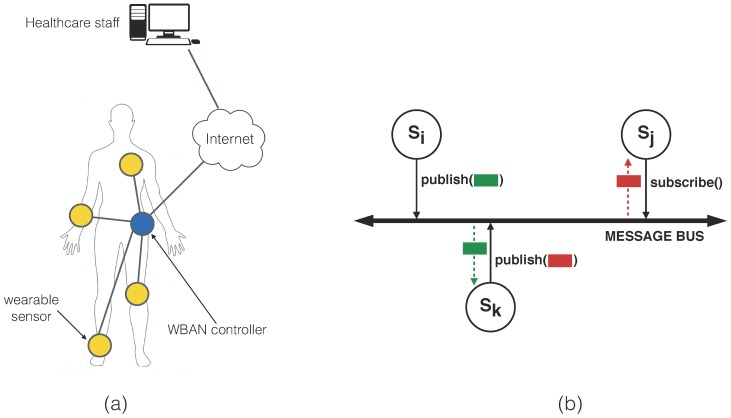
WBAN architecture introduced in [10]: (**a**) physical topology as a network of wearable devices; and (**b**) logically, as a publish-subscribe messaging system (reprinted with permission from the authors).

To better illustrate what a “security policy” may refer to in this context, we will illustrate how our approach can be used together with a fine-grained distributed access control scheme for WBANs, such as the one proposed in [10]. In that work, the authors rely on a lightweight CP-ABE scheme to define secure publish-subscribe protocols for healthcare WBANs (see Figure 2). Data published by each app are encrypted using a CP-ABE policy consisting of a logical AND of the attributes that an entity must possess in order to access the data that it publishes. The authors suggest the use of an LBAC (lattice-based access control) model, which fits well with the idea of using only AND connectives in the policies. Each app in the WBAN must be provided with the appropriate cryptographic keys by a key generation center (KGC). Such keys are derived from the policy attributes (*i.e.*, its access privileges) associated with the app.

In summary, the access policy for an app in a scheme, such as [10], is just a list of attributes A⊆A. Such attributes will be used to derive the app’s cryptographic keys that provide access to data. Furthermore, each app contains a publishing policy consisting of a list of rules that determine which attributes are used to encrypt different types of data or in different contexts.

Another example of what a “security policy” may mean in our context can be found in the user-centric, privacy-preserving permission framework for Android apps described in [11]. Here, the authors propose a permissions model for Android that allows the specification of permissions based on application and system attributes, as well as simple yes or no policies. Examples of such policies provided include “*allow all applications in the group ‘ranked good’ to place calls bypassing the dialer interface*” and “*deny location access to shared app if another app from the same developer has network access*”.

## 3. Similarity Metrics for WBAN Apps

Assume a WBAN and a new app *a* to be deployed on one of its devices. As the first step in the process of deriving a security policy for *a*, we are interested in finding the app $a^{*}$ most similar to *a* within those already in use in the WBAN. This can be achieved by defining an appropriate similarity metric for pairs of apps that somehow takes into account aspects, such as:

- the similarity between the attributes of the two apps, both features and tags;
- the similarity between the set of resources used by both apps according to the attributes of each resource;
- the similarity between the devices where both apps are deployed, considering not only the attributes of both devices, but also the apps already deployed on each one of them; and
- the structural proximity between both apps, this being a measure of how distant in the WBAN one app is from the other considering, for example, the network distance between their host devices or the resources they share.

We next introduce two different random walk-based metrics that compute the similarity between two WBAN apps. We refer the reader to [12] for a brief background on random walk-based similarity metrics for vertices of a graph.

### 3.1. The ALG-2 Metric: Random Walks over Augmented WBAN Structural Graphs

This metric is inspired by recent approaches for computing distances over attributed graphs [9]. It relies on the so-called augmented graph $G^{\prime }=\left(V^{\prime },E^{\prime }\right)$, which is derived from the WBAN structural graph $G_{S}=\left(V_{S},E_{S}\right)$ by adding attribute information in the form of new vertices and edges. First, the set $V_{S}$ is extended with new vertices derived from tags and features. For each tag $t_{j}\in T$, a new vertex $t_{j}$ is added to the set $V_{T}$. In the same way, we build a set of feature vertices $V_{F}$ containing a new vertex $f_{j,k}$ for each possible attribute value. This is computed for all attributes of the three types of elements of the WBAN, so $V_{F}={⋃}_{j=1}^{\left|F\right|}{⋃}_{k=1}^{\left|Dom(f_{j})\right|}f_{j,k}$. Thus, the augmented set of vertices is given by:

$$V^{\prime }=V_{S}\cup V_{T}\cup V_{F}$$

New edges are added to the augmented graph as follows. For each structural vertex $v_{i}$ (*i.e.*, device, app or resource) that has been associated with tag $t_{j}$, a tag-element edge $e_{i,j}=\left(v_{i},t_{j}\right)$ is added to the set $E_{T}$. Similarly, a feature-element edge $e_{i,j,k}=\left(v_{i},f_{j,k}\right)$ is added to the set $E_{F}$ whenever the feature value $f_{j,k}$ is associated with vertex $v_{i}$. Thus, the augmented set of edges is simply:

$$E^{\prime }=E_{S}\cup E_{T}\cup E_{F}$$

**Figure 3:**
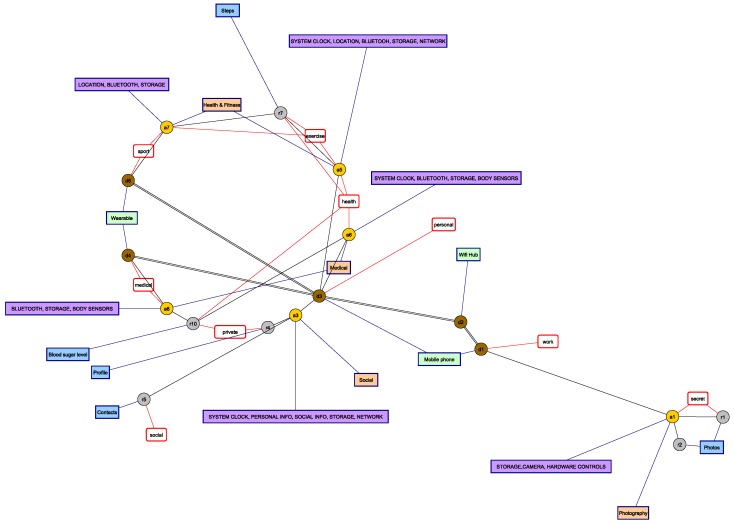
Augmented graph associated with the structural graph shown in Figure 1. Tag and feature vertices are represented by red-lined rounded rectangles and blue-lined rectangles, respectively. Colored feature vertices encode for device types (green), app categories (orange), app permissions (purple) and data types (blue).

Figure 3 shows an example of an augmented graph derived from the structural graph used in Figure 1. Table 1 lists the attributes used to build the augmented graph. Tags are user defined and reflect perceived properties of the WBAN elements. As for attributes, devices are associated with only one attribute (“deviceType”) that captures the type of device. Apps are modeled through two attributes. The first one (“appCategory”) is its category as used in the Google Play Store [13] , while the second models the app permissions. In this case, rather than associating one binary attribute with each permission, we chose to use the notion of permission groups specified in the Reference Android API [14]. Even though in Table 1, we model permissions group as one attribute, in practice, it may be more convenient to consider its values as independent binary attributes. This is motivated by the fact that each app may need permissions belonging to several groups. In such cases, transforming an attribute domain into binary attributes is more efficient than deriving a new domain composed of all possible combinations of the original values. Finally, resources are modeled through one attribute capturing the type of data they contain.

Once the augmented structural graph $G^{\prime }$ is obtained, the similarity between two apps *a* and $a^{\prime }$ can be computed by adapting the state-of-the-art clustering algorithm (SA-cluster) proposed in [9,15]. SA-cluster extends the random walk with restart (RWR) similarity metric proposed in [16] to compute node similarities within node-attributed graphs. The procedure computes short random walks of a given length *l* in the augmented graph $G^{\prime }$. The set of computed distances is then used to cluster the graph nodes. In some application domains, it has been suggested to consider weights for different types of edges. In our case, this would imply using weights $w_{S}$, $w_{T}$ and $w_{F_{j}}$ for the structural, tag and feature edges, respectively.

### 3.2. The ALG-3 Metric: Random Walks over Doubly-Augmented WBAN Structural Graphs

Our second approach is inspired by the procedure described in [17] to compute user similarities in folksonomies. This requires building a doubly-augmented graph $G^{\prime \prime }=\left(V^{\prime \prime },E^{\prime \prime }\right)$ by adding the similarity measures between attribute values to the augmented structural graph $G^{\prime }=\left(V^{\prime },E^{\prime }\right)$ as follows. For each pair of tags $t_{i}$ and $t_{j}$ in $V_{T}$, we add a weighted edge between them $e_{i,j}=\left(t_{i},t_{j},\mathrm{SimTag}\left(t_{i},t_{j}\right)\right)$ where $\mathrm{SimTag}\left(t_{i},t_{j}\right)\in \left[0,1\right]$ is a similarity metric between tags $t_{i}$ and $t_{j}$. The set of inserted edges is denoted $E_{T↔T}$. Similarly, for each pair of feature values $f_{k,i},f_{k,j}\in Dom\left(f_{k}\right)$ corresponding to feature $f_{k}$, we add a weighted edge between them $e_{i,j}=\left(f_{i,k},f_{j,k},\mathrm{SimFeat}\left(f_{k,i},f_{k,j}\right)\right)$ where $\mathrm{SimFeat}\left(f_{k,i},f_{k,j}\right)\in \left[0,1\right]$ is a similarity metric between feature values $f_{k,i}$ and $f_{k,j}$ of attribute $f_{k}$. The set of inserted edges in this case is denoted $E_{F↔F}$. In some cases, it may be convenient to apply a cut-off *ϵ* so that only edges with a similarity above *ϵ* are inserted.

**Figure 4:**
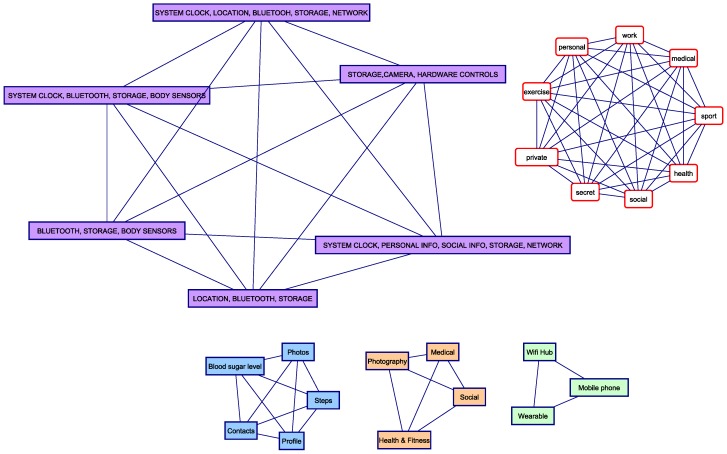
Doubly-augmented structural graph: edges to be added to the graph in Figure 3.

The resulting doubly-augmented graph is $G^{\prime \prime }=\left(V^{\prime \prime },E^{\prime \prime }\right)$, where $V^{\prime \prime }=V^{\prime }$ and $E^{\prime \prime }=E^{\prime }\cup E_{T↔T}\cup E_{F↔F}$. Figure 4 shows the edges that would be added to the augmented graph shown in Figure 3, though no similarity value is shown there.

The similarity between two apps is computed following exactly the same procedure described in Section 3.1, but using the doubly-augmented graph. As before, weighted edges can be used, in which case values for $w_{T↔T}$ and $w_{F_{j}↔F_{j}}$ must be provided in addition to the weights already present in the augmented graph. Some recent works in other domains (e.g., [17]) apply a similar approach. However, our approach differs from [17] in that we insert similarity edges into the attributed augmented graph, while [17] does it directly in the structural graph.

## 4. Evaluation

### 4.1. Description of the Exemplar Case Study: The Smartphone Ecosystem WBAN

**Figure 5:**
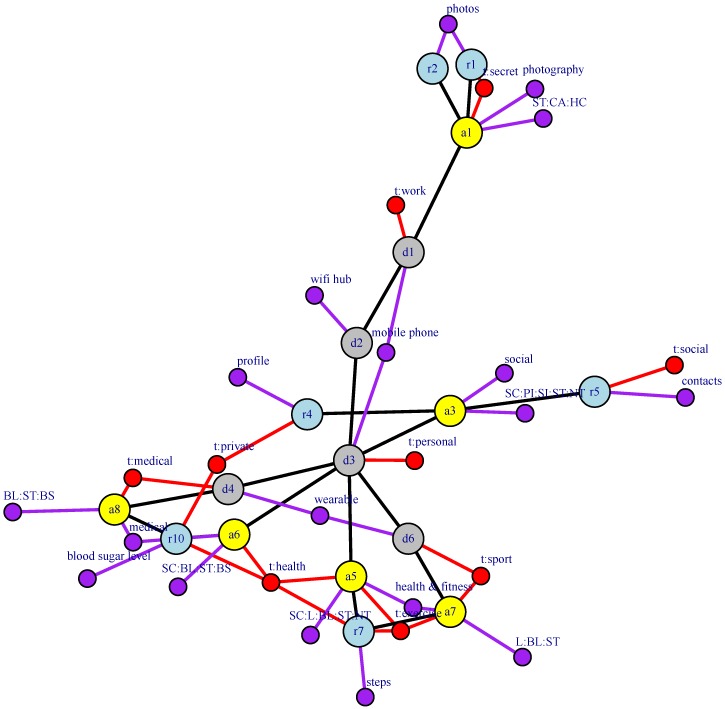
Augmented graph of the smartphone ecosystem use case.

**Figure 6:**
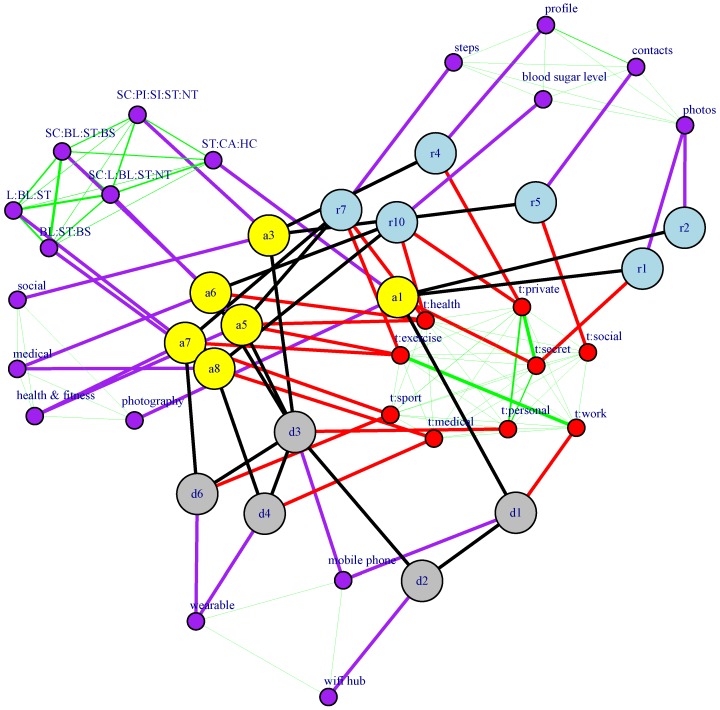
Doubly-augmented graph of the smartphone ecosystem use case.

In this section, we evaluate the two proposed similarity algorithms based on augmented structural graphs by applying them to a smartphone ecosystem example use case (see Figure 5 and Figure 6). In this use case, a user owns two smartphone devices (d1 and d3). Device d1 is a work smartphone, and we consider that a camera application a1 is deployed on it. This application controls two resources r1 and r2 that correspond to pictures having two distinct confidentiality levels. Device d3 is a personal smartphone, and three apps, a3, a5 and a6, are deployed on it. App a3 is a social network application; app a5 is the control app of the fitness monitoring wearable device d6 (which has app a7 deployed on it); and a6 is the control app of the blood sugar monitoring wearable device d4 (which has app a8 deployed on it). Note that paired apps a5↔a7 and a6↔a8 share, respectively, resources r7 and r10 between the controlling app on the mobile phone and the app on the wearable device. In addition to devices d1, d3, d4 and d6, a Wi-Fi hub device d2 is also considered.

### 4.2. Construction of the Augmented Graphs

Four feature types are considered: resource type, app category, device type and app permissions. The first three have been chosen, since it seems likely that users define different privacy policies according to them. For example, a work smartphone may have more restrictive policies than a personal one, and a medical app will generally be seen as more sensitive than a game app. Of course, these perceptions should be tuned by each user. In the same way, the set of permissions requested by an app may influence the user’s perception of how privacy respectful the app is. We have considered as a single feature the whole set of permissions of an app (these sets of permissions are represented by the purple rectangles in Figure 4), instead of taking each permission as an independent feature. The reason is that it seems more logical to compare the similarity between app permission sets than between each permission individually without having a way to assess the privacy risk of each permission. However, if some privacy risk scale for individual app permissions is available, this could be easily leveraged to build a permission similarity based on it. In this case, it might be more interesting to consider each permission as an individual feature.

Similarities between apps permissions have been computed using the well-known Jaccard similarity metric. On the other hand, we compute semantic similarities between tags and between resource type, app category and device type values, as they have a clear semantic meaning for the user. Semantic measures can be broadly divided among knowledge-based, distributional and hybrid semantic measures. Among the available semantic measures, we have chosen to use the extended Lesk (without hyponyms) algorithm, a well-known hybrid semantic relatedness measure [18,19]. The original Lesk algorithm for lexical disambiguation evaluates the similarity between two senses as the number of common words in the definition of the senses in a dictionary [20]. Banerjee and Pedersen in [19] extended the Lesk measure to consider not only the definition of a sense, but also the definitions of related senses through taxonomical links in WordNet [21]. As described in [18], “*WordNet is widely used in natural language processing and computational linguistics. It models the lexical knowledge of native English speakers in a lexical database structured through a semantic network composed of synsets/concepts linked by semantic and lexical relations. In WordNet, concepts are associated to a specific meaning defined in a gloss. They are also characterized by a set of cognitive synonyms (synset) which can be composed of nouns, verbs, adjectives, adverbs.*” Semantic similarities have been computed using the Semilar toolkit 1.0.2 [22] with the algorithm *adapted Lesk-Tanimoto without hyponyms* over the WordNet 3.0 database [23].

**Figure 7:**
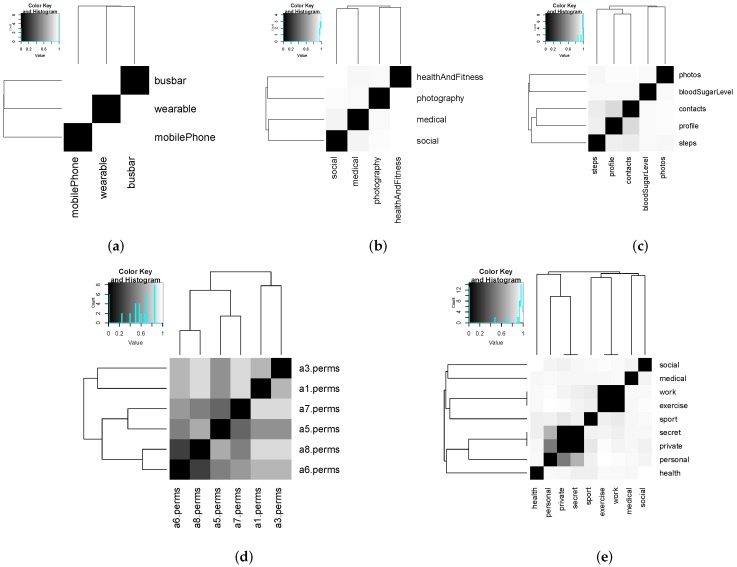
Distances between attribute (tags and features) values of the smartphone ecosystem use case. (**a**) Device type; (**b**) app type; (**c**) resource type; (**d**) permissions set; (**e**) tags.

Figure 7 shows the specific values of distances between all of the tag and feature-value pairs. Note that distances, and not similarity measures, are depicted. Distance is calculated from a similarity value *s* as d=1-s. Results are depicted using heat maps, where each row of the matrix contains the distances of one term (row identifier) to the rest of the terms (column identifiers). For a pair of terms, the whiter the cell is, the less similar the terms are. Conversely, the darker the cell is, the more similar the terms are.

Results in Figure 7 illustrate that similarities between attribute values are rather low in general. Similarities between the app permissions sets are the exception. Notice that a hierarchical clustering algorithm has been applied to the distance matrix before depicting it. For this, we have used the default clustering algorithm implemented by the command hclust in R.

### 4.3. Analysis of Computed Similarities Using Both ALG-2 and ALG-3 Metrics

**Figure 8:**
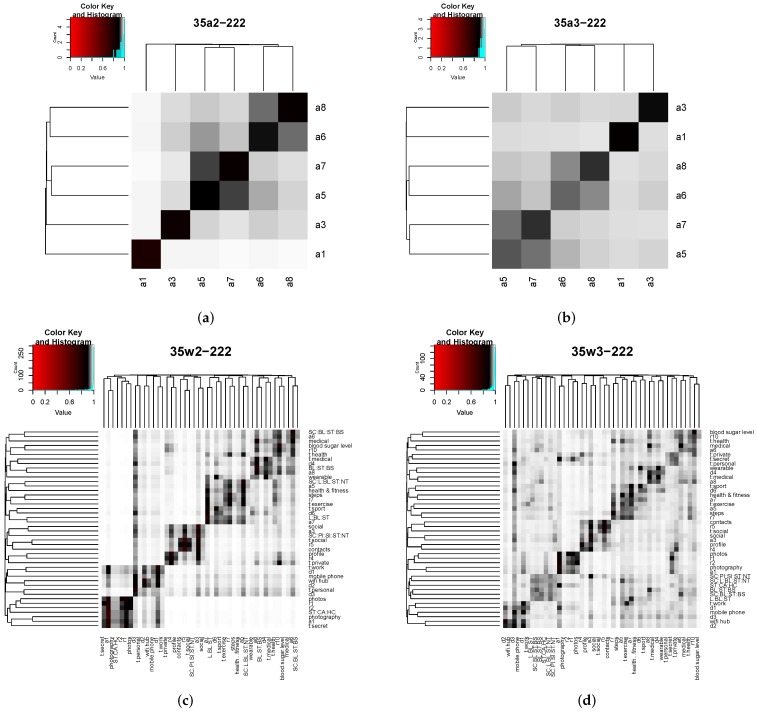
Distances between app nodes and all nodes computed with approaches *ALG-2* and *ALG-3*. The path length is l=35; the restart probability is c=0.15; and structural, tag and feature edge weights are $w_{S}=1$, $w_{F_{j}}=1$ and $w_{T}=1$, respectively. (**a**) App nodes (*ALG-2*); (**b**) app nodes (*ALG-3*); (**c**) all nodes (*ALG-2*); (**d**) all nodes (*ALG-3*).

Figure 8 shows the distances computed with both approaches *ALG-2* and *ALG-3* for the set of app nodes and for all nodes considered in the use case. Both similarity metrics have been implemented in R. Again, heat maps are used to depict results, and the interpretation is the same as in Figure 7: each row contains the distances from one node (row) to the rest of nodes (columns). In this case, two color gradients have been used to facilitate visualization, as most distances fall in the interval $\left[0.8,1\right]$. Preliminary results not shown in this paper suggest that both approaches show a monotonic increasing behavior when length parameter *l* increases, reaching an upper bound when l>25. Thus, we selected a length value of l=35 for the results shown above.

As expected, results obtained with both methods highlight nodes’ structural and attribute closeness (most relevantly, paired apps a6 and a8 or a5 and a7 in Figure 8a,b). However, some differences between both methods can be easily noticed. Firstly, higher similarities (lower distances) with the *ALG-2* approach are somewhat smoothed when using *ALG-3*. Simultaneously, lower similarities (higher distances) with *ALG-2* get increased when using *ALG-3*. This effect can be perceived more clearly by observing the change from Figure 8c to Figure 8d, where the number of white cells decreases significantly (becoming greyish instead), and a part of the darker cells also become greyish.

Additionally, results obtained with *ALG-3* slightly differ from those of *ALG-2* in the resulting clustering. Both methods produce similar clusterings with two key differences: (1) the number of clusters generated by *ALG-3* is larger than obtained through *ALG-2*; and (2), although most of the clusters produced by *ALG-3* are the same as those by *ALG-2*, they lack one or two nodes that have been allocated in two additional clusters by *ALG-3* (one cluster composed of the app permissions sets and another cluster composed of tags secret, personal and private).

In the view of the results achieved by both methods using balanced edge weights, *ALG-3* performs better, as the homogenization of distances combined with the preservation of a meaningful clustering provides more opportunities to find an app similar to the one that needs bootstrapping without requiring both being directly connected through structural links or the same attribute values.

**Figure 9:**
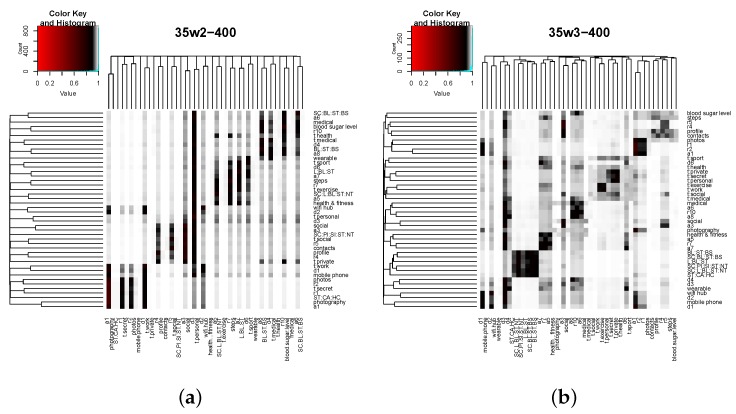

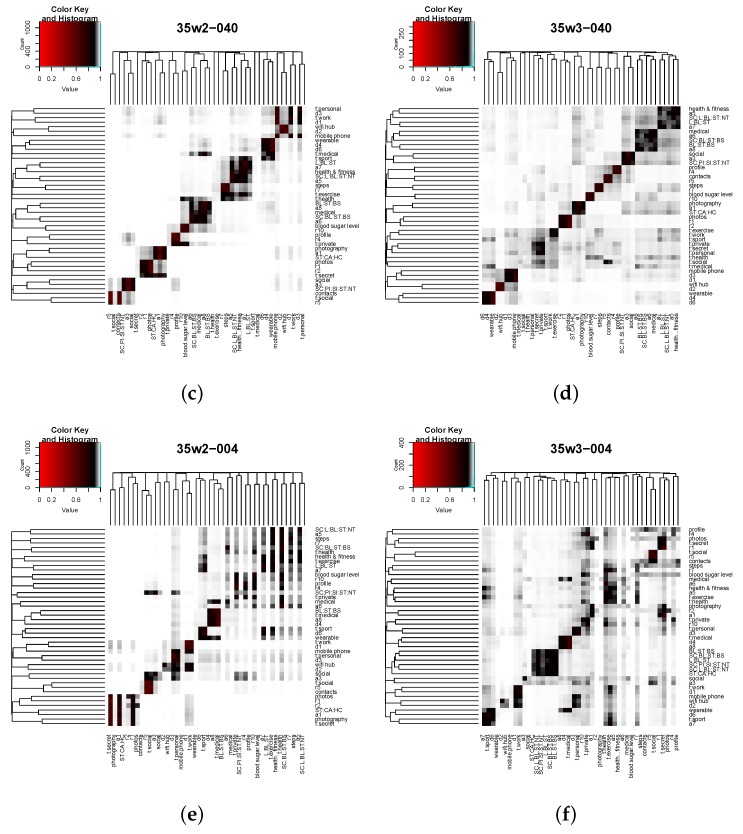
Distances between all nodes computed with approaches *ALG-2* and *ALG-3* and several edge weight combinations. Path length l=35. Restart probability c=0.15. (**a**) *ALG-2* ($w_{S}=2.8$, $w_{F_{j}}=0.1$, $w_{T}=0.1$); (**b**) *ALG-3* ($w_{S}=2.8$, $w_{F_{j}}=0.1$, $w_{T}=0.1$); (**c**) *ALG-2* ($w_{S}=0.1$, $w_{F_{j}}=2.8$, $w_{T}=0.1$); (**d**) *ALG-3* ($w_{S}=0.1$, $w_{F_{j}}=2.8$, $w_{T}=0.1$); (**e**) *ALG-2* ($w_{S}=0.1$, $w_{F_{j}}=0.1$, $w_{T}=2.8$); (**f**) *ALG-3* ($w_{S}=0.1$, $w_{F_{j}}=0.1$, $w_{T}=2.8$).

Lastly, we have analyzed the influence of the different types of edges in both methods by setting different edge weight combinations. Figure 9 shows the results obtained when the weight of one type of edge, among structural, feature and tag types, is set to a significantly higher value than the weight of the other types of edges. Figure 9a,b shows results for edge weights set to $w_{S}=2.8$, $w_{F_{j}}=0.1$ and $w_{T}=0.1$; thus, giving more importance to structural edges. *ALG-2* provides a disperse and non-meaningful clustering, while *ALG-3* gets a slightly enhanced clustering, as, for example, paired apps are grouped together, but with some odd groupings, such as those relating device node d6, which corresponds to a wearable device, with all of the tag nodes, or those relating resource nodes r4 and r5, which correspond to contacts and profile data, with feature nodes steps and bloodSugarLevel.

Figure 9c,d shows results for edge weights set to $w_{S}=0.1$, $w_{F_{j}}=2.8$, $w_{T}=0.1$, giving more importance to feature edges. In this case, both methods *ALG-2* and *ALG-3* obtain meaningful clusterings, with the one provided by *ALG-3* being more accurate as *ALG-2* provides some odd results, such as grouping together feature node mobilePhone with device d2, which corresponds to the Wi-Fi hub, and separating feature node contacts from r5, the resource node with which it is associated.

Finally, Figure 9e,f provides results for edge weights set to $w_{S}=0.1$, $w_{F_{j}}=0.1$ and $w_{T}=2.8$, giving more importance to tag edges. Under this edge weight configuration, both methods get again disperse and quite non-meaningful results, perhaps with the exception of the cluster identified by *ALG-3* that contains the app permissions sets’ nodes.

Among these unbalanced edge weight configurations, the best results are provided by *ALG-3* with weights set to $w_{S}=0.1$, $w_{F_{j}}=2.8$ and $w_{T}=0.1$. However, the obtained result (see Figure 9d) is worse than those provided by both methods under a balanced edge weight configuration (see Figure 8c,d for a comparison), as the unbalanced configuration is, as expected, feature biased, while balanced configurations reflect clusters that take into account all structural and attribute similarity.

## 5. Related Work

We next provide an overview of related works grouped into three main areas: graph-based similarity measures, computing similarity for smartphone apps and the general problem of bootstrapping security and privacy policies.

### 5.1. Graph-Based Similarity Measures

Similarity metrics are widely used today in many application areas. We refer the interested reader to three extensive surveys for a general overview of the variety of existing distance and similarity metrics. First, the Encyclopedia of Distances [24] is an accessible interdisciplinary source that gathers succinct definitions and references for the majority of distances. Second, a more specific comprehensive survey on semantic measures can be found in [18]. Finally, in the last few years, similarity measures in attributed graphs, such as the one proposed in this paper, have attracted much attention in several applications, including community detection, recommendation systems or protein-protein interaction analysis. We refer the reader to [12], a survey on clustering attributed graphs, where graph-based similarity measures similar to those used in this paper are described.

### 5.2. Similarity Measures for Mobile Apps

Several similarity measures for smartphone apps have been proposed over the last few years motivated by two main applications: detecting illegal repackaging and making recommendations.

Android apps are prone to illegal repackaging. This can be done for several reasons, such as stealing ads revenues, software piracy, license evasion or malware injection. Various approaches have been proposed to detect app similarity with this objective: feature based, structure based and program dependency graph based. Section 5.2 of [25] surveys proposals with this goal for Android apps. The works most related to our approach are those that propose similarity measures based on apps’ features [26,27]. They analyze an app code and extract a set of features, such as icons, screenshots, app name, descriptions, requested permissions, used API calls, authorship information, *etc*. Plagiarism between two apps is detected by comparing the extracted features. These feature-based approaches could be easily integrated into our scheme by adding such features to app nodes in the augmented structural graph.

Proposals addressing the problem of app recommendations work quite similarly. Most of them also extract various features from the app information available at the app store, such as its name, developer, description and other users’ reviews [28,29,30,31]. Other proposals extract features from snippets returned by web search engines and from the device logs [32] after running the app. Once apps have been characterized with the extracted features, different approaches are applied to compute similarities between them. For instance, [29,30,32] compute the cosine similarity between the apps’ feature vectors; [32] proposes an algorithm to classify apps based on the latent Dirichlet allocation (LDA) model; and [31] linearly combines the similarity measures computed for each of the apps’ features. The work in [28] substantially differs from previous approaches, as it represents each app according to an app ontology model and then computes semantic similarities between the attribute instances of two sets of apps (the ones in the market and the apps used by the members of a social group) using WordNet. The approach most related to our work is the recommendation system described in [29]. This scheme first computes pair-wise app similarities for all of the apps within the app store and then uses these similarities to build an app similarity graph in which each app is a vertex, and an edge is added only if the similarity between the two apps considered is larger than a certain threshold. To make recommendations, the graph is used to search for the shortest paths having as source and target an app belonging to the set of apps of the user. The apps found within the paths that get a similarity score greater than some specified threshold are candidates for recommendation.

### 5.3. Automatic Inference of Privacy Policies

The problem of automatically learning/inferring security policies has gained relevance in various application domains in which users have been made responsible for creating and administrating such policies. We briefly survey relevant proposals in three dominating areas: website navigation, online social networks and smartphones.

#### 5.3.1. Website Navigation and Online Social Networks

The system proposed in [33,34] helps the user to decide whether or not to accept the privacy policies of visited websites. The user’s previous decisions are stored along with the corresponding P3P privacy policy. When a website offers a new privacy policy that must be accepted in order to enter the site, the system compares the offered privacy policy with the already stored ones using case-based reasoning (CBS) techniques, and depending on the user’s previous decisions, it suggests whether it should be accepted or not.

In the context of location-sharing social networks, the work in [35] proposes the use of machine learning under a user-controlled approach to refine user’s privacy preferences. The system asks the user about his agreement with the system’s decisions to share the user’s location using the already specified policies. Using the feedback provided by the user, the system finds “neighbor” policies to those already specified, such that the satisfaction of user preferences is maximized, and therefore, the user privacy model is refined. The work in [36] bootstraps the location-sharing privacy preferences of a new enrolled user by leveraging the preferences of similar users. Machine learning techniques are used to cluster the whole set of privacy policies used in the social network, and a representative privacy policy is selected for each cluster (privacy policies are assumed to be specified using first-order logic rules that can be compared between them).

In the case of image-sharing social networks, some works have proposed tag-based systems to automatically infer privacy policies for shared images [37,38]. In this domain, users associate tags with images, and based on these tags, image similarity measures are used to infer privacy settings. In [37], the co-owners of a just uploaded image are suggested a bootstrapping privacy policy that corresponds to that of the most similar image (compared to the uploaded one) co-owned by the same set of co-owners. The similarity between images is computed using the tag co-occurrence notion. Similarly, the work in [38] explores the viability of using tags assigned by a certain user to his or her images and the answers of this user regarding his or her preferences for sharing such images with his or her friends. Both items are used to create machine-generated access-control rules that roughly approximate the user’ policy.

Automatic inference of privacy policies in content-sharing social networks has also been approached with machine learning techniques. The works in [39,40,41] apply classifiers to predict whether a post uploaded by a user has a low or a high privacy level. The work in [42] also uses machine learning to classify users into privacy setting classes based on a set of user features. When a new user joins the social network, his or her privacy setting class is inferred based on the privacy settings on the three users most similar to him or her. The system proposed in [43] also identifies groups of similar users, called social circles, using a clustering algorithm that has as input the set of selected user features. The suggestions offered by the system are, as in [42], based on those selected by similar users, but under a more general approach for finding similar items. In [44], which builds upon [43], images uploaded by a user are clustered according to a set of image features. When the user uploads a new image, the system bootstraps it with a privacy policy predicted from the set of most similar images among those already specified by the user, where the most frequent pattern is hierarchically mined following a similar approach to that of [43].

#### 5.3.2. Smartphones

Several recent works reuse some of the approaches proposed for websites and social networks to suggest possible configurations of a smartphone app’s permissions [45,46,47,48,49]. For instance, [45,46] show that it is possible to cluster user preferences regarding the configuration of Android apps’ permissions into a small number of privacy profiles. Some discussion about how to use this result to recommend specific configurations of Android apps’ permission to users depending on their privacy profiles is presented in [46]. The work presented in [47,48,49] goes further and leverages community experience to suggest users with recommendations regarding the configuration of the app’s permissions. In [47], a system called ProtectMyPrivacy (PMP) is proposed. PMP detects access to private data (device identifier, location and address book) at runtime by apps on iOS devices, and depending on the configured user’s preferences, PMP substitutes privacy-sensitive information with anonymized data. Besides, PMP collects users’ decisions regarding whether they have granted or not a permission to an app and uses this information to build a crowdsourced system that makes recommendations to other users by calculating the percentage of users who protect/allow each permission (excluding a 45% to 55% deadband). RecDroid is a system introduced in [48] that assists users to decide whether a permission request should be accepted based on collected requests and responses. The work in [49] leverages friends of the user to assist him or her in setting an app’s privacy policy. Other works that address recommended privacy policy configurations for smartphones focus on using context as an influential factor [50,51,52].

## 6. Conclusions and Future Work

In this paper, we have introduced a framework to compute similarity among apps deployed in a WBAN. Such similarity is based on the attributed structural graph associated with the network and is used to bootstrap new apps with security policies already in use by similar apps. This attempts to guarantee some consistency in the set of deployed security policies while alleviating the load of defining a policy for each new app.

Our scheme is similar to similar approaches proposed in other application domains, such as social networks and folksonomies. In all of these cases, measures of similarity in such complex networks are defined in terms of structural graphs augmented with attributes. In future work, we plan to explore the use of machine learning techniques, particularly classifiers, such as those discussed in Section 5, to automatically infer privacy settings in this domain. On the one hand, assuming that privacy preferences associated with the most similar app $a^{*}$ are offered to the user without modification as the bootstrapping policy for the newly-deployed app *a*, given that similarity is greater than a certain threshold, classifiers may be used to check the accuracy of the suggested policy. However, it is first necessary to carefully design a good set of features for the classifier. On the other hand, classifiers may be used on a simpler setting for inferring an adjusted policy from the *k* apps most similar to the new deployed one.

## Figures and Tables

**Table 1:** Attributes and their domain for the augmented structural graph shown in Figure 3.

Element	Attribute	Domain
–	Tags	personal, work, social, exercise, sport, health, private, medical, secret
Devices	deviceType	wearable, mobilePhone, wifiHub
Apps	appCategory	healthAndFitness, medical, social, photography
	PermGroup	camera(CA), bodySensors(BS), storage(ST), socialInfo(SI), personalInfo(PI), systemClock(SC), hardwareControl(HC), location(L), bluetooth(BT), Network(NT)
Resources	DataType	steps, contacts, profile, bloodSugarLevel, photos
